# Inflammatory Dermatoses: An Audit of Histopathology Reporting Quality and the Role of Clinicopathological Conference

**DOI:** 10.7759/cureus.113775

**Published:** 2026-08-01

**Authors:** Talvinder S Lall, Saroona Haroon

**Affiliations:** 1 Department of Histopathology, King's Mill Hospital, Sherwood Forest Hospitals NHS Foundation Trust, Sutton-in-Ashfield, GBR

**Keywords:** clinical request form information, clinicopathological conference (cpc), dermatology, dermatopathology, histopathology reporting, inflammatory dermatoses, quality improvement, skin biopsy, turnaround time

## Abstract

Background: Inflammatory dermatoses are a frequent indication for skin biopsy and present diagnostic challenges due to overlapping clinical and histopathological features. Accurate diagnosis relies on clinicopathological correlation, often supported by multidisciplinary team (MDT) discussion. The Royal College of Pathologists (RCPath) Tissue pathways for dermatopathology outline national standards for reporting skin biopsies in inflammatory dermatoses. This study aimed to evaluate local compliance with RCPath standards for inflammatory skin biopsy reporting among inflammatory dermatosis cases discussed at skin clinicopathological conference (CPC) meetings.

Methods: A retrospective audit was conducted of all cases discussed at monthly skin CPC meetings between December 2024 and December 2025 (n = 89). Cases with a final histological diagnosis of inflammatory dermatoses were included. Reports were assessed against RCPath-derived standards, including adequacy of request form information, quality of microscopic descriptions, documentation of ancillary investigations and MDT discussion, and turnaround time.

Results: Of 89 skin biopsies discussed at skin CPC meetings between December 2024 and December 2025, 43/89 (48.3%) were identified as inflammatory dermatoses and included in the analysis. Microscopic descriptions met RCPath standards in 43/43 (100%) cases, and ancillary investigations were documented in 27/27 (100%) cases where performed. However, only 8/43 (18.6%) biopsy request forms contained adequate clinical information, defined as documentation of lesion morphology and duration. CPC discussion resulted in diagnostic amendment in 6/43 (14.0%) cases. Turnaround times were prolonged, with only 5/43 (11.6%) reports issued within the seven-day target (mean 44.4 days; median 35 days).

Conclusion: While histopathology report content met RCPath standards, the quality of clinical information provided and prolonged turnaround times represented key areas for service improvement. Improving the completeness of clinical information on biopsy request forms may support more accurate histopathological diagnosis. Skin CPC meetings provide an important forum for diagnostic refinement and clinician education.

## Introduction

Inflammatory dermatoses encompass a broad spectrum of disorders characterized by overlapping histological reaction patterns. Similar microscopic features can occur across clinically distinct conditions, making interpretation of skin biopsies challenging for histopathologists [1,2]. Accurate diagnosis relies on clinicopathological correlation, integrating histological findings with relevant clinical information such as lesion morphology, distribution, duration, and treatment history.

In clinical practice, inflammatory dermatoses are a common indication for dermatology referral and frequently require skin biopsy to establish a definitive diagnosis. Multidisciplinary collaboration between dermatologists and histopathologists plays a central role in this process [3]. Skin clinicopathological conference (CPC) meetings allow for the joint review of clinical and histological findings, facilitating multidisciplinary discussion in diagnostically challenging cases.

The Royal College of Pathologists (RCPath) Tissue pathways for dermatopathology [4] set national standards for the processing and reporting of skin biopsies for inflammatory dermatoses in the United Kingdom. These guidelines define expectations regarding clinical information provided on biopsy request forms, the content of histopathology reports, and turnaround times. Adherence to these standards helps ensure that histopathology reports provide clinically relevant information to support accurate diagnosis, appropriate patient management, and timely clinical decision-making.

Although CPC is a well-established practice in dermatopathology, there are limited published data evaluating compliance with RCPath reporting standards for inflammatory dermatoses in district general hospital settings. These centers provide routine dermatopathology services for a broad patient population. This audit therefore evaluated inflammatory dermatosis cases discussed at monthly skin CPC meetings to assess compliance with these standards, examine the contribution of CPC to diagnostic refinement, and identify opportunities for service improvement.

## Materials and methods

Study design

A retrospective audit was undertaken to evaluate the quality of histopathology reports issued for inflammatory dermatoses in accordance with RCPath guidance. The project was registered through the local clinical audit program.

Case selection

Between December 2024 and December 2025, a total of 89 cases were discussed at monthly skin CPC meetings. Skin CPC serves as the multidisciplinary dermatopathology forum at our institution, where cases requiring specialist discussion are reviewed jointly by dermatologists and histopathologists. Cases were identified from the monthly skin CPC meeting records, and all cases discussed during the study period were screened for eligibility.

All 89 cases were initially screened. In line with the audit objective of evaluating inflammatory dermatoses, only cases with a final diagnosis of an inflammatory dermatosis were included. Neoplastic lesions, cysts, and noninflammatory reactive processes were excluded.

Data collection and audit criteria

Demographic, clinical, and histopathological data were extracted from the CIRDAN laboratory information system (Lisburn, Northern Ireland). Authorized histopathology reports were evaluated using the RCPath Cellular Pathology clinical audit proforma for skin histopathology to assess reporting for inflammatory dermatoses [5], based on the standards outlined in the RCPath Tissue pathways for dermatopathology [4]. The audit criteria assessed three domains: clinical information provided on the biopsy request form, histopathology report content and documentation standards, and turnaround time.

Assessment of clinical information included documentation of lesion morphology and duration, together with the presence of a favored diagnosis or differential diagnosis. Histopathology report content was evaluated according to the documentation of the following RCPath audit criteria: key histopathological features sufficient to support identification of the predominant inflammatory reaction pattern, consideration of clinical differentials in non-specific diagnoses, ancillary investigations, multidisciplinary team discussion, suboptimal biopsy, clinical image review, and collegial discussion.

Turnaround time was defined as the interval between specimen receipt and authorization of the final histopathology report. Where a CPC addendum was issued, the authorization date of the amended report was used. Diagnostic amendments following skin CPC discussion were identified through the presence of an addendum to the original histopathology report. As diagnostic changes following CPC discussion are documented by a formal report addendum in our department, cases without an addendum were considered to have no documented diagnostic amendment.

Compliance targets specified in the RCPath audit proforma were applied to the clinical information and report content domains, with a target compliance of 100%. For turnaround time, RCPath standards specify that at least 80% of reports should be issued within seven days and at least 90% within 10 days [4,5]. Documentation of clinical image review and collegial discussion was assessed descriptively in accordance with principles of good professional practice rather than against predefined compliance targets. Compliance with each audit criterion was assessed by the primary investigator. All assessments were performed by a single reviewer.

Statistical analysis

Data were entered into Microsoft Excel (Microsoft Corporation, Redmond, WA) for descriptive analysis to determine compliance with RCPath standards.

## Results

Case and cohort characteristics

Between December 2024 and December 2025, 89 skin biopsies discussed at monthly CPC meetings were screened. Forty-six cases were excluded as noninflammatory lesions, leaving 43 cases for analysis. The cohort comprised 20 male and 23 female patients, with an overall mean age of 58.3 ± 19.0 years (range 16-104 years). Male patients were older on average than female patients (63.1 ± 21.7 vs. 54.1 ± 15.2 years, respectively).

Distribution of inflammatory dermatoses

Of the 43 included cases, 34/43 (79.1%) received a definitive inflammatory diagnosis, whereas 9/43 (20.9%) were reported as nonspecific inflammation. Definitive diagnoses were stratified according to the dominant histopathological reaction pattern (Table 1). Spongiotic/eczematous-type dermatitis was the most frequently observed pattern, followed by lichenoid/interface dermatitis, neutrophilic dermatoses, and granulomatous dermatoses.

**Table 1:** Distribution of histological diagnoses among cases of inflammatory dermatoses included in the audit

Histological reaction pattern	n (%)
Spongiotic/eczematous	7 (16.3)
Lichenoid/interface dermatitis	6 (14.0)
Neutrophilic dermatoses	5 (11.6)
Granulomatous dermatoses	5 (11.6)
Eosinophilic dermatoses	4 (9.3)
Inflammatory scarring alopecia	3 (7.0)
Ulcerative inflammatory dermatoses	2 (4.7)
Panniculitis	1 (2.3)
Inflammatory sclerosing dermatoses (morphea)	1 (2.3)
Nonspecific inflammatory dermatoses	9 (20.9)
Total	43 (100)

Compliance with RCPath standards

Overall compliance with RCPath standards is summarized in Table 2. Microscopic description and documentation of ancillary investigations demonstrated full compliance with RCPath standards. Although a favored or differential diagnosis was included in most requests, the quality of clinical information on biopsy request forms was limited. Only 8/43 (18.6%) requests documented lesion morphology and duration. Of the remaining 35 cases, 11/35 (31.4%) included clinical or dermatoscopic appearance alone, 14/35 (40.0%) provided a favored or differential diagnosis without a description or duration, and 1/35 (2.9%) contained no relevant clinical information. A neoplastic lesion was listed as the clinical differential diagnosis in 9/35 (25.7%) cases without accompanying descriptive clinical detail.

**Table 2:** Compliance of histopathology reports with RCPath-derived criteria from the tissue pathways for dermatopathology CPC, clinicopathological conference; RCPath, Royal College of Pathologists

RCPath-derived criterion	Compliance, n/N (%)
Clinical request form information	
Documentation of lesion morphology and duration	8/43 (18.6)
Favored or differential diagnosis	39/43 (90.7)
Histopathology report content	
Adequate microscopic description	43/43 (100)
Nonspecific diagnoses addressing clinical differentials (N = 9)	8/9 (88.9)
Documentation of ancillary investigations	27/27 (100)
Documentation of suboptimal biopsy (N = 1)	1/1 (100)
Documentation of collegial discussion	23/43 (53.5)
CPC diagnostic amendment with addendum	6/43 (14.0)
Turnaround time	
Reported within 7 days	5/43 (11.6)
Reported within 10 days	7/43 (16.3)

**Figure 1:**
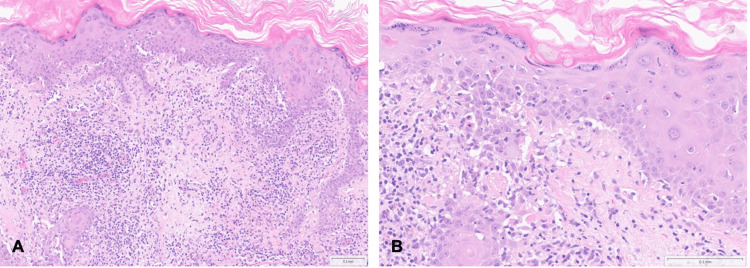
Diagnostic amendment following clinicopathological conference discussion in a case of subacute cutaneous lupus erythematosus The case was initially reported as subacute spongiotic dermatitis with a differential diagnosis of pityriasis rosea, erythema annulare centrifugum, and erythema multiforme. Following clinicopathological conference discussion with review of the clinical history and morphology, subacute cutaneous lupus erythematosus was favored. (A,B) Low- and high-power views demonstrating mild hyperkeratosis and epidermal acanthosis, a superficial perivascular lymphocytic infiltrate, and interface change with lymphocytic infiltration (H&E, ×10 and ×20) H&E: hematoxylin and eosin

**Figure 2:**
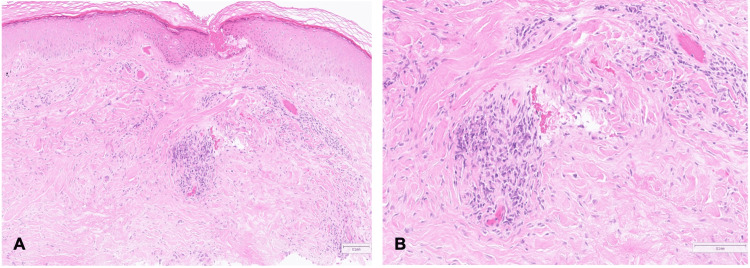
Diagnostic amendment following clinicopathological conference discussion in a case of granuloma annulare The lesion was initially interpreted as a dermatofibroma. Following clinicopathological conference discussion, the features were considered more consistent with granuloma annulare. (A,B) Low- and high-power views demonstrating mild epidermal acanthosis, a dermal fibrohistiocytic proliferation, associated lymphocytic inflammation, and areas of collagen alteration (H&E, ×10 and ×20) H&E: hematoxylin and eosin

Following CPC discussion, the diagnosis was amended in 6/43 cases (14.0%). Clinical images were routinely reviewed during CPC for all cases where appropriate. Representative histological features of selected cases in which the diagnosis was amended following CPC are shown in Figures 1, 2. Turnaround time performance fell below RCPath targets. The mean interval from specimen receipt to report authorization was 44.4 days (median 35 days), with only 5/43 (11.6%) cases reported within seven days.

## Discussion

This first-cycle audit demonstrated strong compliance with RCPath standards for histopathology report content in cases of inflammatory dermatoses discussed at skin CPC meetings. Microscopic descriptions consistently identified appropriate reaction patterns, ancillary investigations were clearly documented, and reports diagnosing nonspecific dermatoses generally addressed the proposed clinical differentials. These findings indicate a high standard of histopathological reporting within our department.

In contrast, adequate documentation of lesion morphology and duration was present in only 8/43 cases (18.6%). The request form is central to the diagnostic pathway in dermatopathology, serving as the primary means of communication between the dermatologist and histopathologist [6]. Provision of essential descriptors such as lesion morphology, distribution, and duration allows histopathological findings to be interpreted in the appropriate clinical context, supporting a more precise diagnosis. In the absence of adequate details, histological examination is limited, increasing the risk of misdiagnoses and potentially delaying patient management [6-8].

Beyond diagnostic accuracy, the completeness of clinical information also influences reporting efficiency. Romano et al. demonstrated that, for inflammatory skin lesions, higher quality clinical information was associated with shorter turnaround times and reduced additional work-up, including further stains or deeper levels [9]. Limited clinical request form detail may delay reporting through the need to review clinical records, communicate with the requesting clinician, or perform additional ancillary testing. As histological patterns in inflammatory dermatoses are frequently nonspecific [1], interpretation depends on assessing histology in parallel with clinical features. Consequently, incomplete request forms may limit the value of the final histopathology report for clinicians.

CPC discussion resulted in diagnostic amendment in 6/43 cases (14.0%). Multidisciplinary discussion is an established component of dermatopathology practice and is particularly valuable in complex or atypical cases, where it improves diagnostic confidence and reduces diagnostic error [10]. Comparable findings have been reported by Wells et al., who found that the diagnosis based on histology alone differed from the final clinicopathological consensus diagnosis in approximately one-quarter of cases, highlighting the value of multidisciplinary clinicopathological review in dermatopathology [11].

Histological patterns in inflammatory dermatoses are commonly shared across multiple conditions, making lesion morphology an important adjunct in establishing an informed diagnosis [2]. In this context, images provide additional information that facilitates correlation between microscopic findings and the dermatological presentation. In our department, clinical images were reviewed alongside histopathological findings during CPC meetings. This approach has been shown to improve clinicopathological concordance in diagnosing inflammatory dermatoses [12].

Turnaround time in histopathology is influenced by multiple factors, notably laboratory workflows and staffing, variability in case complexity, and the need for ancillary investigations [9,13,14]. The turnaround times observed in our cohort did not meet the RCPath recommendations. Many cases included in this audit were reported within a period of significant department backlog, contributing to prolonged intervals from specimen receipt to authorization. Throughout this period, urgent specimens were prioritized over routine biopsies, including those performed for inflammatory dermatoses. Both nationally and globally, histopathology services are under increasing strain due to rising case volumes and workforce shortages [15,16]. While these wider pressures provide important context, the turnaround times observed in this audit were likely influenced by local operational demands and do not represent routine departmental practice.

Following completion of the audit, findings were disseminated through local governance and quality improvement processes. Results were presented at a trust-wide forum attended by senior clinical leadership and were circulated to community dermatology services and general practitioners, given the significant proportion of referrals originating in primary care. The findings were discussed at skin CPC meetings to promote the standardization of request forms to reinforce the importance of documenting lesion morphology, duration, and differential diagnoses. These measures were implemented to support future improvements in reporting quality for inflammatory dermatoses.

This study has several limitations. The relatively small sample size and single-center design may limit the generalizability of the findings. The audit cohort consisted exclusively of cases discussed at skin CPC meetings and may therefore not be representative of all inflammatory skin biopsies reported within the department. Cases selected for CPC discussion are typically diagnostically challenging and may require specialist review or additional investigations. This selection may have contributed to longer turnaround times and may also have influenced the observed reporting compliance compared with routine inflammatory skin biopsies. Furthermore, the audit assessed the documentation and completeness of histopathology reports against RCPath standards rather than independently assessing diagnostic accuracy. As compliance was assessed by a single reviewer, independent verification of the assessments was not performed and may be subject to observer bias.

## Conclusions

Overall, the content of histopathology reports for inflammatory dermatoses discussed at skin CPC meetings showed good compliance with RCPath reporting standards. However, clinical information provided on biopsy request forms was often inadequate, and turnaround times did not meet RCPath standards and were influenced by a period of significant departmental backlog. Improving the completeness of clinical information may facilitate clinicopathological correlation and support histopathological interpretation. Skin CPC meetings provide an important platform for multidisciplinary discussion, clinician education, and promoting good biopsy request practices. The findings of this audit informed targeted service improvement initiatives, including communication of results across secondary and primary care services and promotion of standardized biopsy request form completion. Future reaudit will be important to close the audit loop, evaluate the effectiveness of these interventions, and monitor ongoing adherence to RCPath standards.
